# Magnitude of psychological distress and associated factors among war survivor women in Northern, Ethiopia, 2022: a community-based cross-sectional survey

**DOI:** 10.1186/s12905-024-03161-5

**Published:** 2024-06-20

**Authors:** Endalamaw Salelew, Yohannes Awoke Assefa, Rediet Getachew, Goshu Nenko, Biruk Fanta, Tadele Amare, Demeke Demilew, Endalkachew Dellie, Andualem Yalew Aschalew, Geta Asrade, Zelalem Demeke, Kegnie Shitu, Getachew Azeze Eriku, Chanyalew Worku, Alemu Kassaw Kibret, Tsegaye Gebremedhin Haile, Melkamu Tamir Hunegnaw, Haileab Fekadu, Ayenew Molla, Aysheshim Kassahun Belew, Asmamaw Atnafu, Tadesse Guadu, Mezgebu Yitayal, Netsanet Worku, Kassahun Alemu Gelaye, Telake Azale, Tadesse Awoke

**Affiliations:** 1 Department of psychiatry, School of Medicine, University of Gondar, Gondar, Ethiopia; 2 Department of Epidemiology and Biostatics, Institute of Public Health, University of Gondar, Gondar, Ethiopia; 3 Department of Health system and Policy, Institute of Public Health, University of Gondar, Gondar, Ethiopia; 4 Department of Environmental and Occupational Health and Safety, Institute of Public Health, University of Gondar, Gondar, Ethiopia; 5 Department of Health Promotion and Health Behavior, Institute of Public Health, University of Gondar, Gondar, Ethiopia; 6 Department of occupational therapy, School of Medicine, University of Gondar, Gondar, Ethiopia; 7 Department of Human Nutrition, Institute of Public Health, University of Gondar, Gondar, Ethiopia; 8 Department of Physiotherapy, School of Medicine, University of Gondar, Gondar, Ethiopia; 9 Department of Medical Nursing, School of Nursing, University of Gondar, Gondar, Ethiopia; 10 School of Health Sciences, Western Sydney University, Campbell town, Sydney, NSW 2560, Australia

**Keywords:** War, Women mental health, Community, Psychological distress, Northern Ethiopia

## Abstract

**Background:**

Civilian war and internal conflicts increase the incidences of mental health conditions among war survivors. It is crucial to assess war-related psychological consequences in war-affected areas in Ethiopia to intervene in the future. Thus, this study aimed to determine the magnitude of psychological distress and associated factors of psychological distress among war survivor women in Northern, Ethiopia.

**Methods:**

A community-based cross-sectional survey was conducted, and 1596 war survivor women were recruited to participate using a face-to-face interviews with a census sampling technique from May 1–30, 2022. The psychological distress was assessed using a Kessler psychological distress scale (K10). Bi-variable and multi-variable logistic regression analyses were used, and variables with a *p*-value less than 0.05 in the multivariable analyses were considered statistically significant.

**Result:**

In this study, the response rate was 100% and the prevalence of psychological distress was 44.90% at a 95% CI: (42.40, 47.40). Psychological distress was significantly associated with the education of ability to read and write (AOR = 2.92; 95% CI: 2.12, 4.01), primary education and above (AOR = 3.08; 95% CI: 2.09, 4.54), housewife (AOR = 5.07; 95%CI: 2.64, 9.74), farmer (AOR = 8.92; 95%CI: 4.03, 19.70), emotional violence (AOR = 1.52; 95%CI: 1.05, 2.18), physical violence (AOR = 3.85; 95%CI: 2.37, 6.26) and sexual violence (AOR = 3.25; 95%CI: 1.98, 5.33) whereas being separate was protective for psychological distress (AOR = 0.38; 95%CI: 0.16, 0.92).

**Conclusion:**

The prevalence of psychological distress was found to be high. Therefore, women who are housewives, married, farmers, educated, and who have experienced violence must be the focus of governmental and private collaborative interventions to prevent war-related psychological morbidity and mortality.

## Introduction

Armed conflicts are complex, dynamic and multi-factorial phenomena. Their onset, intensity and duration are driven by a wide range of factors [1, 2]. Civilian wars and internal conflicts in a country increase the incidences of mental health problems among survivors, such as post-traumatic stress disorder (PTSD), depression, anxiety, psychosis, substance use, and neurological disorders [2, 3]. Emotional suffering related to war may occur not only due to direct exposure to life-threatening situations and violence, but also through indirect stressors, such as injury to or death, malnutrition, illness of relatives or caregivers, economic hardships, geographic displacement, security constraints, and continuous disruptions of daily living [4, 5]. According to a systematic review and meta-analysis report on the updated World Health Organization (WHO) prevalence figures, the prevalence of mental illnesses (schizophrenia, depression, anxiety, bipolar disorder, and PTSD) in the populations impacted by conflict was 22.1% at post-conflict moment [6]. In conflict-affected areas women are vulnerable for mental health conditions due to violence, stigmatization, lacks of social and physical capital, and healthcare [7–9]. Some studies showed that the prevalence of depression among women in post-conflict areas were, 40.2% in Syria [10], 47.4 in South Sudan [11], 58.73% in Juba southern Sudan [12], 57% in Congo female refugees [13], 38.1% in Swat, Pakistan [14], 44% in Burundi [13], 31% in Darfur Sudan [15] and post-traumatic stress disorder among war survivor women was 29.9% in Syrian refugees [10], 42.54% in South Sudan [12] and 73% in Congo female refugees [13]. Previous research works revealed that the prevalence of mental health problems were high in conflict-affected areas. For instance, depression 51.6–82,1% [16, 17], mental distress 59.4% [18] and perceived stress 76.1% [19], and post-traumatic stress disorder ranges 34.5 − 59.8% [20–24].

Untreated mental health disorders and psychosocial problems in the post-war areas result in suicidality and disruption of brain development, affecting children’s learning and behavioral development [25]. For instance, a disproportionate number of war-affected people reported suicidal ideation and attempt ranging from 0.17 to 70.6% and 0.14 to 15.1% in studies across the globe, respectively [26] and 65% suicidal ideation in Congo refugees [13]. Interpersonal sensitivity may be a key mechanism contributing to psychopathology following trauma [27]. There is a high correlation between mothers’ and children’s vulnerability to the psychological consequences of war [3]. Furthermore, women are at higher risk of violence, intimidation, or arbitrary detention perpetrated because of their actual or perceived role in the conflict, and they are the wives, mothers, daughters or whose relatives are fighting [28].

A study carried out among war-survivors revealed that sexual abuse as adult civilians were (77.0%) and as children were (52.6%) [29]. Most notably, a variety of symptoms were experienced by respondents of sexual and gender-based violence, feelings of humiliation (91.5%), insomnia (72.8%), confusion and embarrassment (70.6%), feelings of hatred (37.4%), frustration (28.6%), fear and worries about the future (26.7%), floating anxiety (29.4%), feelings of rejection (23.5%), and a sense of powerlessness (22.1%) [29].

This indicates that the psychological response to an injury or disabilities consists of complex traumatic reactions [30]. That means early treatment for the invisible wounds of war, are critical in ensuring adequate recovery and rehabilitation in their living settings [30–34]. Mental health rehabilitation therapy is crucial to reduce physical and emotional traumas and restore hope among psychologically affected survivors [33], such as narrative exposure therapy and interpersonal therapy for post-war in low-resource settings [35].

The armed conflict in the northern part of Ethiopia caused widespread adverse effects on many innocent people, such as injuries, disabilities, internal displacement, familial and financial losses, interruptions to social support systems, disruptions to healthcare services and other aspects. Women and children are suffered significantly with mental health consequences of the war, and post-war mental healthcare should be given priority [36]. There are lacks of data regarding the levels of psychological distress and risk factors that are associated with psychological distress among women who were living in the North Gondar zone of Ethiopia, which is heavily affected by armed conflict. Almost all African nations lack post-conflict mental health legislation, despite the rising rates of anxiety, depression, and post-traumatic stress disorder [37]. These highly complex settings need an integral to establish psychosocial rehabilitation to reduce levels of physical and emotional traumas and improve social functioning through individual and community interventions. As a result, this study was aimed to assess the prevalence and associated factors of psychological distress among war-survivor women in north Gondar zone, Ethiopia for future intervention.

## Methods and materials

### Study setting and population

This study was conducted using a community-based cross-sectional survey in the north Gondar zone of Amhara, Ethiopia from May 1 to 30/2022. The north Gondar zone capital city Debark, is 822.6 Km road distance from the country’s capital. This zone has eight districts and the three districts were affected by the war, particularly Dabat, Chenna, and Zarima were severely affected areas. There were three internally displaced people sites (IDPs) in Dabat, Debark and Zarima with more than ten thousand people residing. In each camp, the central government, local and international nongovernmental organizations were working to address basic services and security including mental health psycho-social support needs and well-being. Likewise, the University of Gondar was the leading among governmental institutions by providing complementary mental health and psycho-social support in the war-affected areas. The study populations were women aged eighteen and above, excluding women with hearing and communication problems and who were living in politically insecure areas.

### Sample size determination and technique

The three severely war-affected kebeles Dabat, Chenna, and Zarima were selected, and proportional allocation was applied for each selected kebele based on the number households (HHs) using the updated post-conflict Community Health Information System (CHIS) register. Then, interviews with the women were conducted from home to home. If more than one eligible respondent are available in one HH, one eligible woman was randomly selected to be interviewed. Therefore, in this survey a total of (*n* = 1596) women were recruited to participate using a census sampling technique.

### Study variables

Psychological distress was considered as dependent variable whereas socio-demographic characteristics such as (age, marital status, occupation, educational status, pregnancy status, and family size), gender-based violence such as (emotional, physical and sexual), post-traumatic stress disorder, and suicidal behavior were included in the independent variables.

### Data collection procedures and tools

The data were collected by 12 Bachelor of Science in Psychiatry nursing working at nearby primary healthcare settings and supervised by three masters’ professionals in clinical and community mental health. Data were collected using interviewer-administered with Amharic versions of the questionnaire for a month. Initially, the questionnaire was prepared in English, and translated to the local language (Amharic), and then translated back to English to ensure its consistency and accuracy. Two days intensive training was given for data collectors and supervisors on how to interview, handling ethical issues, securing respondents’ informed written consent for participation, maintaining privacy and confidentiality with the Kobo tools a week before the actual data collection. The tool was pre-tested on 5% of the sample 80 women in Woqen kebele to ensure the internal validity of the study. The internal consistency of the tool was good with the Cronbach’s alpha of 0.82.

The measurement had five sections; socio-demographic, psychological distress, post-traumatic stress disorder, gender-based violence and suicidal behaviors. The psychological distress was assessed using a Kessler psychological distress scale (K10), which involves ten questions about emotional states, each with a five-level response rate. Each item is rated from “1” none of the time to “5” all of the time with a minimum and a maximum score of 10 and 50 respectively. Individuals were labeled based on their scores such as 10–19 were likely to be well, 20–24 were likely to have a mild mental disorder, 25–29 were likely to have a moderate mental illness, and 30–50 were likely to have a severe mental illness [38]. This screening tool’s psychometric and factor structure was evaluated among adults in Ethiopia and revealed that K-10 could effectively assess psychological distress with a good internal consistency of Cronbach’s alpha of 0.83 [39].

The probable post-traumatic stress disorder was assessed with primary care PTSD screening for DSM-5. (PC PTSD − 5) is a 5-item screening designed to identify individuals with probable PTSD in primary care settings. If a respondent endorses a traumatic exposure, they can score a 0–5 on the PC-PTSD-5, a count of “yes” to the questions about how the trauma has affected them in the past month. For women, a cut-point of 4 resulted in many false negatives. The data were collected following the end of the war in the area. As a result, a cutoff point of 3 and above was considered to minimize the false negative [40].

Violence against women was also assessed using the WHO multi-country study on women’s health and domestic violence against women questionnaire, which has 13-item and measures three violence domains. This questionnaire was used for a survey in 10 countries representing diverse cultural settings including in Ethiopia. Of these four questions for psychological violence, six for physical violence and three for sexual violence. Answering ‘yes’ to any question from 13-item, considered there was violence against women and responding ‘yes’ to any question to each domain verified as physical, emotional and sexual violence against women [41]. This measurement tool was validated in northern and southern Brazil and showed that the Cronbach’s alpha coefficients of 0.88 and 0.89 respectively [42]. In this study, the internal consistency was excellent with Cronbach’s alpha of 0.85.

### Data processing and analysis

The collected data were checked for the completeness and consistency by the supervisors, primary investigator and research teams daily. The final data were downloaded from the Kobo tool and analyzed with the Statistical Package for Social Sciences (SPSS) version 20. Descriptive statistics were calculated to explain the socio demographic, post-traumatic stress disorder, gender based violence, suicidal behaviors and psychological distress with frequency, percentage, tables and graphs. In the binary logistic regression analysis, bi-variable and multi-variable analyses were performed to show the associations between independent variables and outcome variable. Variables with a *p*-value of less than 0.25 at bi-variable logistic regression analysis were candidates for multi-variable logistic regression analyses. The independent variables that scored a *p*-value of less than 0.05 at multi-variable logistic regression analyses were considered as statistically significant. The strengths of association were described with an adjusted odds ratio and 95% confidence interval. Model fitness was checked by Hosmer-Lemeshow statistics and revealed a chi-square value of 5.098 with significance at a *p*-value of 0.576, which means that the model has a good fit. There is no any co-linearity among independent variables because the variance inflation factor (VIF) was below 2 for each candidate variable.

## Results

### Socio-demographic characteristics of study participants

A total of 1596 participants were interviewed, with a response rate of 100%. In this study, the mean age of the participants was (M = 36.1, SD = 11.8) with a minimum and maximum age of 18 and 85 respectively. Of the participants, 96.4% were orthodox Christian religious followers. The majority of the participants, 1360 (85.2%) were married, 1128 (70.7%) were unable to read and write, 1430(89.6%) were housewives occupationally, 891(55.8%) had 1–5 family size and 1125 (70.5%) had no under-five children (Table 1).

**Table 1:** Socio-demographic characteristics of the study participants (*n* = 1596)

Variables	Category	Frequency	Percent
Age	Mean (SD)	36.1(± 11.8)	
Religion	Orthodox	1539	96.4
	Muslim	57	3.6
Marital status	Married	1360	85.2
	Single	79	4.9
	Divorced	67	4.2
	Widowed	49	3.1
	Separate	41	2.6
Maternal education status	Unable to read and write	1128	70.7
	Able to read and write**	286	17.9
	Primary education	168	10.5
	Others*	14	0.9
Occupation	Housewife	1430	89.6
	Farmer	86	5.4
	Daily laborer	48	3.0
	Student	17	1.1
	Employee	6	0.4
	Other	9	0.6
Family size	Number of families 1–5	891	55.8
	Number of families > 5	705	44.2
Pregnancy status	No	1434	89.8
	Yes	162	10.2
Having under-five children	No	1125	70.5
	Yes	471	29.5

Others* *secondary school and above*, Able to read and write** *women having informal educations such as religious education and community based adult education.*

### Prevalence of post-traumatic stress disorder

In this study, all the participants experienced the traumatic situation which was the war four months ago. Out of these participants, 204 (12.8%) had shown probable post-traumatic stress disorder symptoms, according to a primary care post-traumatic stress disorder screening for DSM-5 at a cutoff point ≥ 3 in the past month.

### Prevalence of violence against women

In this study, a significant proportion of the study participants, 466(29.2%) reported violence against women. Of which, 209 (13.1%), 127 (8.0%), and 130 (8.1%) encountered emotional, physical, and sexual violence respectively (Table 2).

**Table 2:** Prevalence of gender-based violence among the study participants in the war affected community, (*n* = 1596)

Variables	Frequency	Percentage
Emotional violence		
No Yes	1387	86.9
	209	13.1
Physical violence		
No Yes	1469	92.0
	127	8.0
Sexual violence		
No Yes	1466	91.9
	130	8.1

### Prevalence of suicidal behavior among the study participants

Concerning to suicidal behaviors, 68(4.3%), and 29(1.9%) of the participants reported suicidal ideation, and suicide attempt in the past month respectively (Table 3).

**Table 3:** Prevalence of suicidal behavior among the study participants in the war affected area, (*n* = 1596)

Sno	Variables	Category	Frequency	Percentage
1.	Ever suicidal ideation	No	1501	96.7
		Yes	95	6.0
2.	Suicidal ideation in the last 1 month	No	1528	95.7
		Yes	68	4.3
3.	Ever suicidal plan	No	1560	97.7
		Yes	36	2.3
4.	Ever suicide attempt	No	1560	97.7
		Yes	36	2.3
5.	Suicide attempt in the last 1 month	No	1567	99.2
		Yes	29	1.8

### Prevalence of psychological distress

**Fig. 1:**
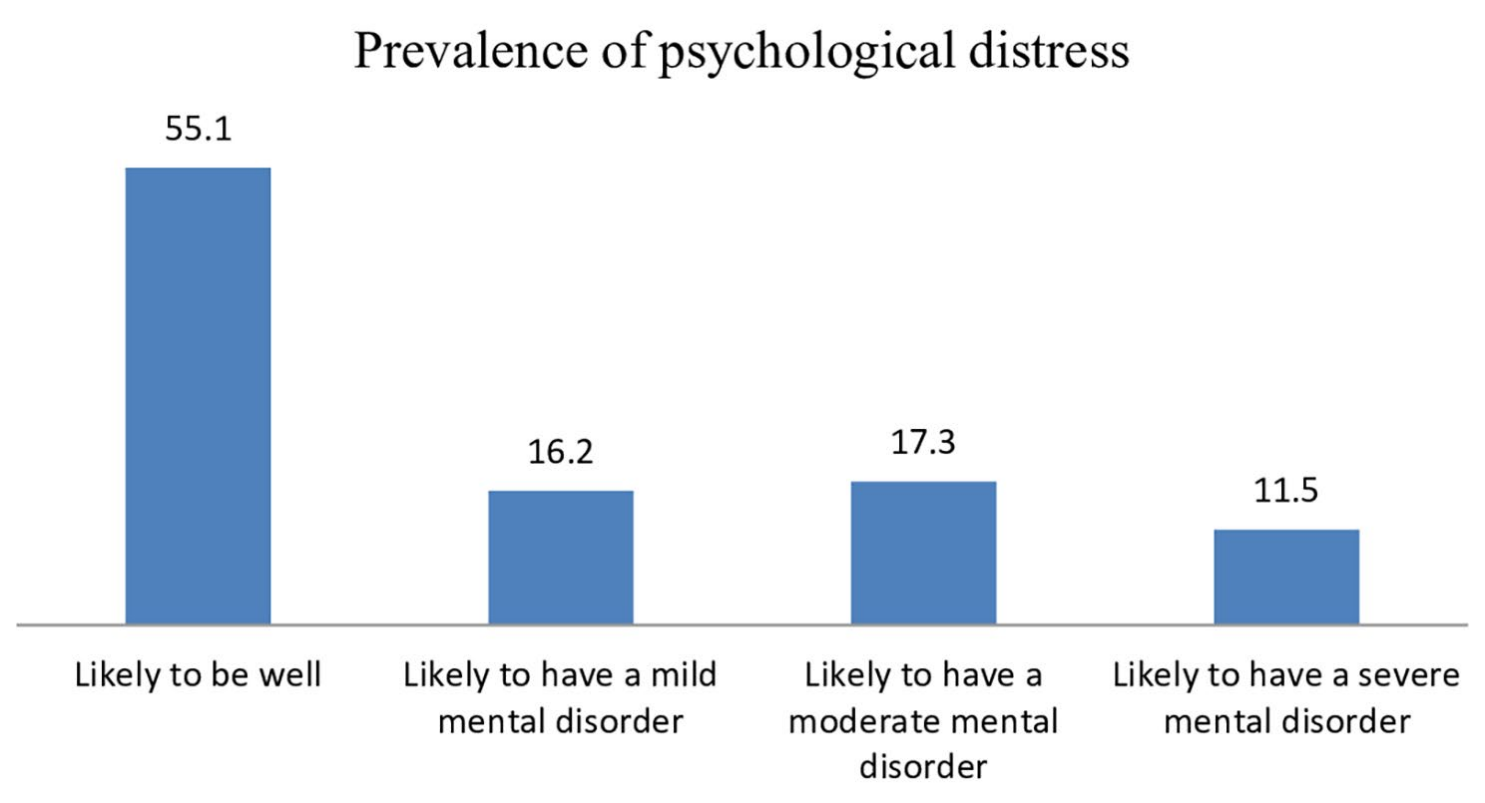
Prevalence of psychological distress among study participants in the community survey, (*n* = 1596)

In this community survey, the magnitudes of psychological distress were assessed using Kessler-10 and revealed that a significant proportion, 717 (44.9%), had a 95%CI (42.4, 47.4). Of the participants with psychological distress, 258 (16.2%), 276 (17.3%), and 183 (11.5%) had mild, moderate, and severe mental disorders, respectively Fig. 1.

### Factors associated with psychological distress

In the bi-variable logistic regression analysis variables, age, marital status, educational status, occupation, having under-five children, pregnancy status, PTSD, emotional, physical, and sexual violence, and an attempt were associated with psychological distress at P - values of less than 0.25.

The multivariable logistic regression analysis showed that marital status, educational status, occupation, emotional violence, physical violence, and sexual violence were significantly associated with psychological distress at a *p*-value less than 0.05.

In this study, the risk of developing psychological distress among participants with a marital status of separate is reduced by 62% as compared to married participants (adjusted odds ratio (AOD): 0.38; 95% CI: 0.16, 0.92). The study revealed that the educational status of being able to read and write, primary education and above, increased the risk of developing psychological distress by about three times as compared with participants who were unable to read and write in education (AOD: 2.92; 95% CI: 2.12, 4.01), and (AOD: 3.08; 95% CI: 2.09, 4.54), respectively.

This research found that the occupation of a housewife and farmer had a 5 and 9 times higher likelihood of developing psychological distress as compared to other jobs (such as being a student, employed, or merchant) (AOD: 5.07; 95%CI: 2.64, 9.74) and (AOD: 8.92; 95%CI: 4.03, 19.70), respectively.

On the other hand, survivors of gender-based violence were more likely to develop psychological distress as compared to those not experiencing gender-based violence. For instance, emotional violence about two times, physical violence about four times, and sexual violence more than three times more likely to lead to psychological distress as compared to their counterparts at (AOD: 1.52; 95%CI: 1.05, 2.18), (AOD: 3.85; 95%CI: 2.37, 6.26) and (AOD: 3.25; 95%CI: 1.98, 5.33) respectively (Table 4).

**Table 4:** Bivariate and multivariate logistic regression analysis of factors associated with psychological distress among study participants, (*n* = 1596)

Variables	Category	Psychological distress		COD(95% CI)	AOD (95% CI)
		No, *n* (%)	Yes *n* (%)		
Age	Mean(SD)	36.1(± 11.8)		0.99(0.95,1.05**)**	1.01(0.94,1.09)
Marital status	Married	738(54.3)	622(45.7)	1	1
	Single	44(55.7)	35(44.3)	1.42(0.85,2.35)	1.03(0.56,1.87)
	Divorced	42(62.7)	25(37.3)	2.37(1.07,5.25)	1.58(0.65,3.86)
	Widowed	38(77.6)	11(22.4)	1.34(0.69,2.60)	1.35(0.63,2.86)
	Separate	17(41.5)	24(58.5)	0.49(0.21,1.12)	0.38\(0.16,0.92)*
Education status	Unable to read and write	707(62.7)	421(37.3)	1	1
	Able to read and write	100(35.0)	186(65.0)	0.32(0.24,0.42)	2.92(2.12,4.01)***
	Primary education and above	72(39.6)	110(60.4)	0.82(0.56,1.21)	3.08(2.09,4.54)***
Occupation	Housewife	780(54.5)	651(45.5)	2.55(1.52,4.26)	5.07(2.64,9.74)***
	Farmer	38(45.2)	46(54.8)	3.69(1.90,7.16)	8.92(4.03,19.70)***
	Others**	61(75.3)	20(24.7)	1	1
Probable PTSD	No	785(56.4)	607(44.6)	1	1
	Yes	94(46.0)	110(54.0)	1.51(1.11,3.62)	1.35(0.96,2.90)
Pregnancy	No	787(54.9)	647(45.1)	1	
	Yes	92(56.8)	70(43.2)	1.08(0.78,1.50)	1.08(0.71,1.64)
Having under-five children	No	670(55.7)	455(44.3)	1	1
	Yes	209(44.4)	262(55.6)	1.84(0.87,1.33)	1.53(0.69,1.24)
Emotional violence	No	809(58.3)	578(41.7)	1	1
	Yes	70(33.5)	139(66.5)	2.78(2.05,3.78)	1.52(1.05,2.18)**
Physical violence	No	852(58.0)	617(42.0)	1	1
	Yes	27(21.3)	100(78.7)	5.11(3.30,3.92)	3.85(2.37,6.26)***
Sexual violence	No	855(58.3)	611(41.7)	1	1
	Yes	24(18.5)	106(81.5)	6.18(3.92,9.74)	3.25(1.98,5.33)***
Ever suicide attempt	No	871(55.8)	689(44.2)	1	1
	Yes	8(22.2)	28(77.8)	4.42(2.00, 9.77)	1.22(0.42,3.58)

Note: Significance of association* <0.05, **<0.01 and ***<0.001, 1 reference, Crude odds ratio (COD), Adjusted odds ratio (AOD), Confidence interval (CI)

Others**: *occupation includes student, employed, merchant, daily laborers*

## Discussion

The present community-based cross-sectional survey serves as an overview of the incidences and associated factors of psychological distress among war survivor women in Ethiopia. According to the findings, 44.9% of study participants with a 95% confidence interval of 42.40 to 47.40, experience psychological distress. This result is consistent with research conducted among people affected by armed conflict in Burundi (44%) [13], South Sudan (47.4%) [43], and in two different studies conducted in Syria, 40.2% [10], and 46.9% [44]. However, the findings of this study indicate that the magnitude of psychological distress is greater than the findings in Darfur, Sudan, among conflict-affected women (31%), in Pakistan (38.1%) [15], in Pakistan (38.1%) [45], in Sri Lanka (18.8%) [46], and in Syria (30.5%) among refugees with residence permission in Germany [47], and (22.1%) in a new World Health Organization’s prevalence estimates of mental disorders in conflict settings [3]. The discrepancy could be caused by differences in study populations, measuring instruments, study methodologies, and participant sociocultural variations. Moreover, studies in Pakistan, Sri Lanka, and Syria focused on female refugees residing in wealthy countries with access to social and practical aid services, whereas this study included women who were living in war-affected areas just after the conflict where basic services are disrupted.

On the other hand, the results of this study are lower than the findings from Ethiopia, in Tigray (82.1%) [17], in conflict-affected areas during the COVID-19 era, (59.8%) [18], and another study in north Shewa, (76.1%) [19], the Democratic Republic of Congo 57% among female refugees [48], and in Juba, southern Sudan (58.73%) [12]. The possible explanations for the differences might be the diverse sociocultural background of the study populations. The fact that the probability of acquiring mental health conditions is high in situations where there is a lack of security and basic services. For displaced persons living in their own and adjacent nations, it can be challenging to offer basic services and safeguards in low-income countries. Furthermore, studies conducted in Ethiopia during the time when double-burden stressors (conflict and COVID-19) existed, as well as in Tigray Ethiopia, suggested that the long-lasting war might increase the risk of depression.

In this study, separated women were less likely to have psychological distress as compared to married women. This is supported by a study carried out in South Sudan among conflict-affected people [44]. The possible justification might be that married women are at risk of violence due to their husbands’ actual or perceived involvement in fighting [28]. In addition, in the northern part of Ethiopian cultural experiences women are submissive to their husbands. Thus, being separate may provide relief from such stressful and traumatic experiences.

The other astonishing finding of this study was that the educational status of being able to read and write and above was significantly associated with psychological distress as compared to being unable to write. This is contrary to the idea that educational status is directly related to better mental health [49, 50]. The possible justification could be that those experiencing terrible news are at risk of mental illness because people able to read and write and those with primary education and above are near social media and fortune to get such awful news. These findings are unexpected and need further study in the future.

The odds of occupational status for housewives and farmers were higher as compared to other occupations (students, employed, merchants, and daily laborers). This is supported by previous research showing that housewives are at increased risk of mental illness because they are overburdened with household responsibilities and have low social visibility [51]. The possible explanation for the association might be due to the most common problem in war-affected areas, which are financial insecurities that may lead housewives to experience low self-esteem. On the other hand, the higher risk of psychological distress among farmer occupations might be due to an increased risk of financial insecurity when working in politically insecure environments.

In this study, physical, emotional, and sexual violence were significantly associated with psychological distress. This is supported by previous research works across the globe, which reported women who had been exposed to physical, emotional, and sexual violence are in significantly worse psychological distress as compared to unexposed women [52–54].

This study, despite its capacity to provide valid evidence about war-related mental health conditions, has some limitations. Since the data were collected through face-to-face interviews, participants might have been hiding sensitive issues and inclined to reply in ways favorable to others by either underreporting or overreporting. Moreover, the cross-sectional design we used has prevented us from reporting casual-effect relationships.

## Conclusion

In this study, the prevalence of psychological distress among women in war-affected areas was found to be high. In spite of personal vulnerability, mental health conditions are significantly worse due to the exposure to trauma and violence among women living in war conflict-affected areas. Because of this, women who are housewives, married, farmers educated and who have experienced violence must be the focus of governmental and private collaborative interventions. Additionally, it is strongly advised to conduct more research on resilience, coping strategies, and cause and effect correlations.

## Data Availability

The datasets used in this study are available from the corresponding author on reasonable requests.

## Abbreviations

AOD: Adjusted odds ratio
CI: Confidence interval
COR: Crude odds ratio
DSM-5: Diagnostic and statistical manual of mental disorders
PC-PTSD: Primary care post-traumatic stress disorder
SD: Standard deviation
SPSS: Statistical package for social sciences
WHO: World health organization
